# Incidence of Anxiety in Latest Life and Risk Factors. Results of the AgeCoDe/AgeQualiDe Study

**DOI:** 10.3390/ijerph182312786

**Published:** 2021-12-03

**Authors:** Franziska Dinah Welzel, Melanie Luppa, Alexander Pabst, Michael Pentzek, Angela Fuchs, Dagmar Weeg, Horst Bickel, Siegfried Weyerer, Jochen Werle, Birgitt Wiese, Anke Oey, Christian Brettschneider, Hans-Helmut König, Kathrin Heser, Hendrik van den Bussche, Marion Eisele, Wolfgang Maier, Martin Scherer, Michael Wagner, Steffi G. Riedel-Heller

**Affiliations:** 1 Institute of Social Medicine, Occupational Health and Public Health (ISAP), Medical Faculty, University of Leipzig, 04103 Leipzig, Germany; Melanie.Luppa@medizin.uni-leipzig.de (M.L.); Alexander.Pabst@medizin.uni-leipzig.de (A.P.); Steffi.Riedel-Heller@medizin.uni-leipzig.de (S.G.R.-H.); 2 Institute of General Practice, Medical Faculty, Heinrich-Heine-University Düsseldorf, 40225 Düsseldorf, Germany; Pentzek@med.uni-duesseldorf.de (M.P.); Angela.Fuchs@med.uni-duesseldorf.de (A.F.); 3 Department of Psychiatry, Technical University of Munich, 81675 Munich, Germany; DWeeg@schoen-klinik.de (D.W.); horst.bickel@tum.de (H.B.); 4 Central Institute of Mental Health, Medical Faculty Mannheim, Heidelberg University, 68159 Mannheim, Germany; Siegfried.Weyerer@zi-mannheim.de (S.W.); jochenwerle@yahoo.de (J.W.); 5 Institute for General Practice, Hannover Medical School, 30625 Hannover, Germany; Wiese.Birgitt@mh-hannover.de (B.W.); Oey.Anke@mh-hannover.de (A.O.); 6 Department of Health Economics and Health Services Research, University Medical Centre Hamburg-Eppendorf, 20246 Hamburg, Germany; c.brettschneider@uke.de (C.B.); h.koenig@uke.de (H.-H.K.); 7 Department of Neurodegenerative Diseases and Geriatric Psychiatry, University Hospital Bonn, 53127 Bonn, Germany; Kathrin.Heser@ukbonn.de (K.H.); wolfgang.maier@ukb.uni-bonn.de (W.M.); michael.wagner@uni-bonn.de (M.W.); 8 Department of Primary Medical Care, Center for Psychosocial Medicine, University Medical Center Hamburg-Eppendorf, 20246 Hamburg, Germany; bussche@uke.uni-hamburg.de (H.v.d.B.); meisele@uke.uni-hamburg.de (M.E.); m.scherer@uke.de (M.S.); 9 German Center for Neurodegenerative Diseases (DZNE), 53127 Bonn, Germany

**Keywords:** incidence, anxiety, late life, cohort study

## Abstract

Research on anxiety in oldest-old individuals is scarce. Specifically, incidence studies based on large community samples are lacking. The objective of this study is to assess age- and gender-specific incidence rates in a large sample of oldest-old individuals and to identify potential risk factors. The study included data from N = 702 adults aged 81 to 97 years. Anxiety symptoms were identified using the short form of the Geriatric Anxiety Inventory (GAI-SF). Associations of potential risk factors with anxiety incidence were analyzed using Cox proportional hazard models. Out of the N = 702 older adults, N = 77 individuals developed anxiety symptoms during the follow-up period. The incidence rate was 51.3 (95% CI: 41.2–64.1) per 1000 person-years in the overall sample, compared to 58.5 (95% CI: 43.2–72.4) in women and 37.3 (95% CI: 23.6–58.3) in men. Multivariable analysis showed an association of subjective memory complaints (HR: 2.03, 95% CI: 1.16–3.57) and depressive symptoms (HR: 3.20, 95% CI: 1.46–7.01) with incident anxiety in the follow-up. Incident anxiety is highly common in late life. Depressive symptoms and subjective memory complaints are major risk factors of new episodes. Incident anxiety appears to be a response to subjective memory complaints independent of depressive symptoms.

## 1. Introduction

Anxiety in old age is a frequent mental health problem [1,2,3,4] with an estimated point prevalence of 17% for any anxiety disorder [5]. Specific phobias (10%), followed by generalized anxiety disorder (4%) and social phobia (4%), have been found to be the most prevalent anxiety disorders in oldest-old individuals [5]. The burden of anxiety in later life is extensive including impairments in quality of life [6], limitations in daily activities [3,6], increased risk of developing dementia [7], and an excess in health-care costs [8]. Some studies have linked anxiety, specifically generalized anxiety disorder, with increased mortality in women [9]. However, research on the association of anxiety and mortality rate is rather inconclusive [10,11]. Besides anxiety disorders, sub-threshold anxiety symptoms are highly prevalent in late life [1,12] and have been associated with self-reported functional decline in older adults [13]. Still, anxiety in late life has been reported to be a largely unrecognized and underestimated cause of suffering in older adults [14]. 

While knowledge about the prevalence of anxiety in the oldest-old helps to recognize the extent of a mental health issue and the need for treatment at a given point in time, information on the incidence of anxiety is important to understand long-term developments and major drivers of new episodes of late-life anxiety. So far, only few studies on incident anxiety in late life are based on large community samples [15]. Previous cohort studies found incidence rates varying between 0.8 per 1000 person-years up to 32 per 1000 person-years for various anxiety disorders [16,17,18,19]. However, most prior studies reporting on the incidence of anxiety in older people either did not include individuals aged 80 years and above [16,18,19], assessed the incidence of anxiety only in specific subgroups of older people (e.g., spouses of dementia or visually impaired individuals) [20,21,22] or did not use anxiety measures specifically developed for older adults [16,20,21]. Other studies relied on primary care or computer recorded data for anxiety without further specification on how those anxiety diagnoses have been assessed [23,24]. So far, the literature particularly lacks information on gender- and age-specific incidence rates of anxiety symptoms in the group of oldest-old individuals using anxiety measures explicitly developed for this age group. Diagnostic instruments used to assess anxiety in younger age groups may not be as adequate to measure anxiety in late life as the presentation of anxiety symptoms change in late life [25]. Further, loss experiences (i.e., the death of a related person) as a possible risk factor for incident anxiety in late life have been rarely considered in the past. In a prior study we found prevalent anxiety symptoms to be associated with recent loss experiences, next to depression and cognitive function [1]. Similarly, other studies reported an increase in anxiety in older adults post bereavement [26,27]. Therefore, recent experiences of bereavement may be a significant risk factor for the occurrence of subsequent anxiety in late life. In sum, there is clearly a need for more research on anxiety, specifically incidence of anxiety symptoms, in the oldest-old.

Thus, the objectives of this study are (a) to determine age- and gender-specific incidence rates of anxiety symptoms in a primary care sample of oldest-old individuals (81+ years) and (b) to identify risk factors for incident anxiety symptoms and describe the course of incident anxiety. 

## 2. Materials and Methods

### 2.1. Study Population

Data were derived from the study “Needs, Health Service Use, Costs and Health-Related Quality of Life in a Large Sample of Oldest-Old Primary Care Patients (85+) (AgeQualiDe)” and its preceding study the “German Study on Ageing, Cognition, and Dementia in Primary Care Patients (AgeCoDe)”. The AgeCoDe and AgeQualiDe studies are multicenter prospective cohort studies conducted in six study centers in Germany: Hamburg, Bonn, Düsseldorf, Leipzig, Mannheim, and Munich. The initial recruitment of 3327 patients for the AgeCoDe study was realized via 138 participating general practitioners (GPs). Participants were regular primary care patients aged 75 years or over and with absence of dementia at baseline. Data were collected through structured clinical interviews conducted in participants’ homes with baseline and six follow-up assessments (FU) conducted every 18 months from 2003 until 2013. The AgeQualiDe study continued the AgeCoDe study with three additional FU assessments every 10 to 15 months until 2017. The present study uses data from FU waves five (FU5) and six (FU6) of the AgeCoDe study (2010–2014) and FU wave seven of the AgeQualiDe study (2014–2015) assessing the same patient group. These FU waves were chosen because only in this period anxiety symptoms were assessed. The AgeCoDe and AgeQualiDe study design and eligibility criteria for the AgeCoDe cohort at baseline have been described in detail elsewhere [28,29]. 

**Figure 1:**
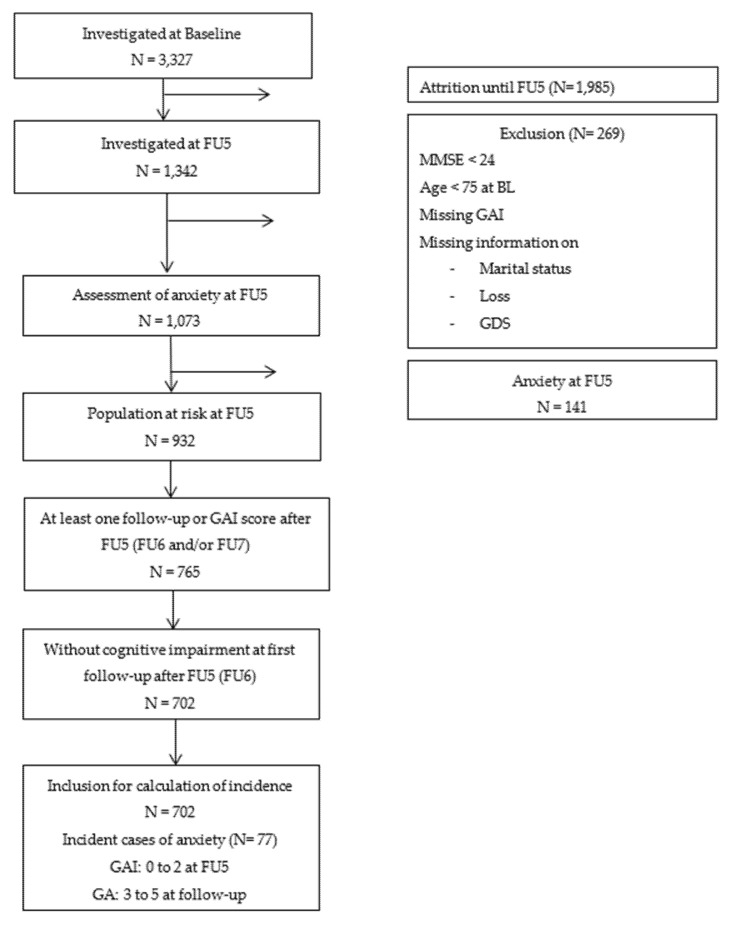
Flow chart for sample selection at baseline and follow-up.

Of the N = 3327 participants at baseline, a total of N = 1342 were investigated at FU5. Of those, N = 269 individuals were excluded due to incomplete assessments, not meeting inclusion criteria at baseline (age < 75) or having cognitive impairment (scoring less than 24 on the Mini Mental State Examination, MMSE [30]) at FU5. Furthermore, individuals with cut-off anxiety symptoms at FU5 were excluded from the analysis (N = 141) leaving a population at risk of N = 932. For final inclusion in the analyses of incident anxiety, individuals needed to have at least one assessment of anxiety symptoms at the subsequent follow-ups (FU6 and/or FU7) and having no cognitive impairment at FU6 (≥24 on the MMSE). Information on the sampling frame and the number of participants at baseline and FU for the present study is given in Figure 1. 

### 2.2. Ethical Consideration

Study conduct was in accordance with the Declaration of Helsinki and approved by the local ethic committees of all six participating study centers (Hamburg: OB/08/02, 2817/2007, MC-390/13; Bonn: 050/02; 174/02, 258/07, 369/13; Mannheim: 0226.4/2002, 2007-253E-MA, 2013-662 N-MA; Leipzig: 143/2002, 309/2007, 333-1318112013; Düsseldorf: 2079/2002,2999/2008, 2999; München: 713/02, 713/02 E). Prior to study participation, written informed consent was provided by all patients and/or their proxies.

### 2.3. Measures

#### 2.3.1. Geriatric Anxiety Symptoms

Anxiety symptoms were identified using the short form of the Geriatric Anxiety Inventory (GAI-SF) [31]. The GAI-SF comprises five items on a yes/no response scale assessing the severity of anxiety symptoms. The GAI-SF has good psychometric properties and has been proposed as a screening instrument for generalized anxiety disorder in older people [31,32]. A cut-off score of ≥3 was used to identify individuals with increased symptoms of anxiety [33] and possible anxiety disorder [31]. The same cut-off score has been used previously in other studies assessing anxiety in late life [1,29,33]. The GAI-SF is an instrument specifically suitable to assess anxiety in late life due to its brevity, the simple response format, and the absence of somatic symptom items. Anxiety was assessed initially at FU5 (for exclusion of cases with GAI-SF ≥ 3) and for observation at follow-ups 6 and 7.

#### 2.3.2. Predictor Variables

Potential predictors of incident anxiety were assessed at study FU5. Sociodemographic variables included gender, age, marital status (single, married, divorced/widowed), living situation (alone, not alone), and education (high, middle, low) according to the new CASMIN educational classification (Comparative Analysis of Social Mobility in Industrial Nations) [34]. 

Loss experiences were defined as cases of death in the closer social environment of participants within the last 18 months. No limitations on the type of relationship were imposed. 

Depressive symptoms were measured with the 15-item Geriatric Depression Scale (GDS-15) [35]. The GDS-15 consists of a yes/no response format and has been developed for specific use in older adults. The instrument has been shown to have good psychometric properties [36]. A cut-off score of ≥6 was used in the present study to differentiate between people with and without depressive symptoms [37].

Subjective memory complaints (SMC) were assessed using two items adapted from Geerlings et al. [38] by asking participants if they have noticed a decline in their memory (“Do you feel like your memory has become worse?”) and if so, whether they were concerned about it (“no”, “yes, that is a worry”, “yes, that is a serious worry”). Participants with no subjective memory decline and those who were not concerned about subjective memory impairments were categorized as having no SMC. Participants who considered their subjective memory decline as worrisome were regarded as having SMC [38].

Functional impairments were measured on the level of mobility, hearing, and eyesight. Participants were asked whether they experienced no, some, significant, or most severe difficulties in each domain. Participants reporting difficulties were considered impaired in the respective domain.

### 2.4. Statistical Analyses

Incidence of anxiety is presented as incidence rates (IR) with 95% confidence intervals, and was calculated as the number of new cases of anxiety at follow-up waves six/seven divided by person-years at risk. In cases of new episodes of anxiety symptoms during the observed follow-up period the first time of appearance was estimated as half way between the last assessment without anxiety (GAI < 3) and the first assessment with cut-off anxiety (GAI ≥ 3). IRs are presented as total incidence. Total incidence refers to individuals who develop a new anxiety episode during the observation period. In contrast, life time incidence or first time incidence refers to those who develop anxiety for the first time in their life [39]. Individuals without anxiety onset during the observed follow-up period were censored at the end of the study period (FU7). Furthermore, individuals were censored in case of death or occurrence of impaired general cognitive function (Mini Mental State Examination test (MMSE) < 24, [30]). Multivariable cox proportional hazard regression models were performed in order to analyze the strengths of associations between incident anxiety symptoms and predictor variables. The cox regression is suitable in order to model the simultaneous influence of several predictor variables on the time-to-event. Predictor variables included in the regression model were age, gender, previous loss experience, depressive symptoms, subjective memory complaints, and functional impairments. GP practices were included as a cluster variable in a complex sample regression model in order to incorporate the sample design in the analysis and achieve adjusted estimated standard errors. Requirements for the cox proportional hazards regression model were tested beforehand by checking Schoenfeld residuals for each risk factor and applying the ph test (testing for proportional hazards) for the overall cox regression model. Due to a strong overlap of depression and anxiety, regression analyses were run separately for the overall sample and a subsample of individuals with no depression at FU5. Kaplan–Meier curves were analyzed to assess time to incident anxiety symptoms. Data analyses were performed using SPSS 25 Windows (SPSS Inc., Chicago, IL, USA). The level of statistical significance was set to *p* < 0.05.

## 3. Results

### 3.1. Study Population

Of the 702 participants included in the analyses without anxiety at follow-up five, 66.4% were female (N = 466) and 33.6% (N = 236) were male. The study sample was on average 86.4 (SD = 2.8) years old, and more frequently widowed or divorced (N = 445, 63.4%) than married (N = 211, 30.1%) or single (N = 46, 6.5%). About half of the study sample had a low educational level (N = 381, 54.3%) and was living alone (N = 371, 52.8%). Table 1 shows the characteristics of the study sample at follow-up five. Individuals investigated at FU5 and excluded from the analyses (N = 640) did not significantly differ from the study sample with regard to marital status (66.0% vs. 63.4% widowed/divorced, 26.5% vs. 30.1% married, 7.5% vs. 6.6% single, χ^2^ (2, 1340) = 2.298, *p* = 0.332). However, excluded individuals were older (M = 87.2, SD = 3.47 vs. M = 86.4, SD = 2.82, *p* < 0.001), more often female (70.8% vs. 66.4%, χ^2^ (1, 1342) = 3.002, *p* = 0.047) and had a lower educational level (61.4% vs. 54.3% low education, 29.4% vs. 30.3% medium education, 9.2% vs. 15.4% high education, χ^2^ (2, 1342) = 13.286, *p* = 0.001) compared to included participants. Sample characteristics of excluded participants can be found in the supplementary material (Table S1).

### 3.2. Age- and Gender-Specific Incidence Rates of Anxiety Symptoms

Table 2 shows the age- and gender-specific incidence rates of anxiety symptoms in the sample of oldest-old primary care patients. In total, 77 participants (11%) developed incident anxiety symptoms (GAI-SF: 3+ points) during the follow-up period (FU6/FU7). In the overall sample, the incidence rate of anxiety was 51.3 (95% CI: 41.2–64.1) per 1000 person-years. Women had a higher incidence rate (IR: 58.5, 95% CI: 43.2–72.4) compared to men (IR: 37.3, 95% CI: 23.6–58.3). Overall, the incidence rate decreased with age from 57.4 (95% CI: 41.8–78.6) in the youngest age group (81 to 85 years) to 52.0 (95% CI: 35.7–72.8) in the intermediate age group (86 to 90 years) to 20.1 (95% CI: 6.7–62.4) in the oldest age group (91+ years). However, the highest incidence rate was found in women aged 86 to 90 years old (IR: 66.8, 95% CI: 47.2–94.6).

### 3.3. Risk Factors of Incident Anxiety Symptoms

**Figure 2:**
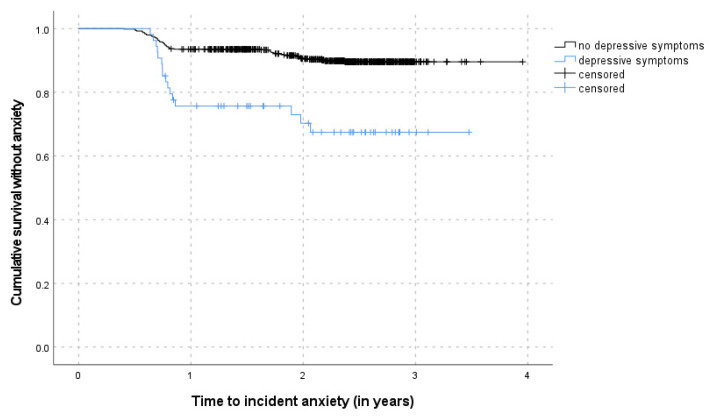
Kaplan–Meier survival curves according to depressive symptoms.

**Figure 3:**
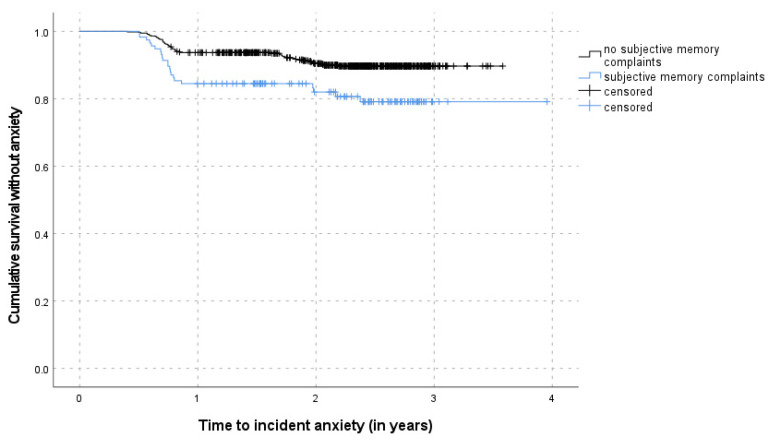
Kaplan–Meier survival curves according to subjective memory complaints.

Table 3 shows the results of the multivariable Cox proportional hazards regression of time to incident anxiety adjusted for the cluster-effect of the recruiting GPs for the overall sample and the subsample without depression at FU5, separately. Depression and subjective memory complaints were positively associated with incident anxiety symptoms in the overall model. The risk for incident anxiety during the follow-up period was significantly increased among individuals with depressive symptoms at FU5 (HR: 3.20, 95% CI: 1.46–7.01). Similarly, the presence of SMC at FU5 increased the risk of incident anxiety symptoms in the follow-up twice as much compared to those without previous SMC (HR: 2.03, 95% CI: 1.16–3.57). The interaction term of depressive symptoms and SMC was not significant in the overall model. The predictive value of SMC for incident anxiety was further confirmed by the multivariable analysis of the subsample without depression at FU5 (HR: 1.92, 95% CI: 1.09–3.39) indicating depression and SMC as independent risk factors for the development of subsequent anxiety symptoms. Figure 2 and Figure 3 show the Kaplan–Meier survival curves according to status of depression and SMC, respectively. Age, gender, recent loss experiences, and functional impairments were not significantly associated with incident anxiety.

### 3.4. Course of Anxiety Symptoms

Table 4 shows the course of anxiety from follow-up waves 5 to 7. With regard to the overall sample, a minority (2.4%) of participants developed incident anxiety symptoms that persisted through follow-up wave seven. Of those with incident anxiety, about one fifth (22.1%) experienced persistent anxiety. Both, temporary anxiety symptoms (5.3% vs. 13.7%, chi2 (1, 559) = 5.739, *p* = 0.027) and persistent anxiety symptoms (5.3% vs. 29.4%, chi2 (1, 525) = 16.688, *p* = 0.002) were associated with previous depressive symptoms at FU5. While preceding depressive symptoms were more frequent in those with persistent anxiety (29.4%) compared to individuals with temporary anxiety (13.7%), the difference between temporary anxiety and persistent anxiety with regard to preceding depression was not significant (chi2 (1, 68) = 2.159, *p* = 0.136). Sample characteristics according to the course of anxiety are shown in Table 5.

## 4. Discussion

The aim of the present study was to assess age- and gender-specific incidence rates of anxiety symptoms in a primary care sample of oldest-old individuals (81+), to identify risk factors and to describe the course of incident anxiety symptoms. There was a considerable rate of incident anxiety symptoms of 51.3 (95% CI: 41.2–64.1) per 1000 person-years in the overall sample. The incidence rate was higher in women compared to men (58.5 vs. 37.3) and decreased with age from 57.4 in the youngest age group (81 to 85 years) to 20.1 in the oldest age group (91+ years). Multivariable analysis showed presence of subjective memory complaints and depressive symptoms to be predictive of incident anxiety in the follow-up. Neither gender, age, recent loss experiences nor functional impairments were significantly associated with incident anxiety symptoms in a multivariable model. Of those individuals with incident anxiety, more than one-fifth (22.1%) experienced persistent anxiety in the course of two follow-up waves.

### 4.1. Incident Anxiety Symptoms in Late Life

Epidemiological studies reporting on incident anxiety in late life are rare. Previous studies reported incidence rates for various anxiety disorders ranging from 0.8 to 32 in older adults [16,17,18,19,40]. However, most studies either assessed first time incidence of anxiety retrospectively [16,40,41], which is prone to recall bias, and/or reported results only for younger age groups of older adults [18,19,40]. Little is known about the incidence of anxiety symptoms in community dwelling cohorts of oldest-old individuals. Thus, comparing the results of our study to previous literature is difficult. A study from the UK assessing entries from a primary care database reported a much lower incidence rate for anxiety symptoms of 5.4 per 1000 person-years for individuals aged 75+ years [24]. A reason for this difference in incidence rates may lie in differences with regard to study design, sample age and anxiety assessment. On the one hand, the present study assessed anxiety symptoms using an age-appropriate screening instrument for anxiety; on the other hand, Walters et al. [24] analyzed GP recorded entries in a database over a period of 10 years. The study by Walters et al. [24] only included older adults who were examined for anxiety symptoms by their attending GP. However, recognition of mental health problems has been shown to be challenging in primary care [42]. Generally, there seems to be an under-detection of mental disorders by GPs [43,44]. Another barrier to the detection of incident anxiety in primary care may lie in the reluctance of older adults to report milder symptoms of anxiety [45,46]. Thus, analyses based on GP recorded database entries will likely lead to an underestimation of incident anxiety in late life. Another study assessed incident anxiety symptoms in late life using population-based age-specific screenings over a course of two years [12]. In comparison to our study, Kang et al. [12] found a higher proportion of incident anxiety symptoms for older adults in general (65+ years) and oldest-old individuals specifically (80+ years) of 29.2% and 44%, respectively. However, comparisons based on incidence proportion may be less meaningful than comparison of incidence rates, as they do not consider the person-time at risk. This is specifically challenging when comparing studies with varying survey periods. In sum, our results show that incident anxiety symptoms are highly common in late life, which is roughly in line with the few previous studies in this field. 

### 4.2. Risk Factors for Incident Anxiety in Late Life

With regard to predictors of incident anxiety in late life, the results of the present study indicate two important risk factors. The first is depression which is a well-known comorbidity of anxiety and is frequently discussed in terms of concept overlap [32] and timeline of disease onset [47,48]. The present data indicate depressive symptoms to precede subsequent anxiety symptoms in latest life. While one previous study [41] did not find major depression at baseline to be associated with incident anxiety disorders in a sample of adults aged 60+ years; at large, our finding is in line with other studies showing depression as an important risk factor for the development of subsequent anxiety [17,20,49]. The results of the present study do not allow drawing conclusions on the true timeline of onset with regard to depression and anxiety, as the total incidence of anxiety symptoms has been assessed instead of first-time incidence. It is therefore possible that the first onset of anxiety and depression may have occurred at some point earlier in life. Nevertheless, the results of this study do suggest that awareness for the possibility of subsequent anxiety should increase in the presence of current depressive symptoms in oldest-old individuals.

Subjective memory complaints were found to be the second risk factor for incident anxiety in the follow-up period. Previous literature has frequently reported an inverse relationship of anxiety with cognitive performance [50,51,52]. The association between anxiety and diminished cognitive performance has been discussed in terms of a two-way relationship. On the one hand, anxiety may function as a marker and prodromal symptom of cognitive decline in later life [53,54,55]. On the other hand, higher cortisol levels as well as attentional shifts during states of anxiety have been shown to compromise cognitive performance [51,56,57]. Further research showed comorbid depression mediating this relationship of anxiety with cognitive performance [58]. In the present study, we looked at the influence of SMC on incident anxiety symptoms. SMC is highly common in late life [59] and has been connected to mild cognitive impairment [60], depression [59], and anxiety [61] in the past. The results of this study indicate SMC to be predictive of incident anxiety symptoms in a sample of overall cognitively healthy oldest-old individuals without depression. Our results therefore suggest that SMC is a risk factor for incident anxiety independent of depression in latest life and support the notion of anxiety symptoms as a response to perceived memory difficulties.

In contrast to our assumption, previous recent loss experiences are not associated with incident anxiety symptoms in a sample of oldest-old individuals. While anxiety has been described as a natural and possibly clinically significant response to bereavement [62], the loss of a relative or a close friend may not be in general a good predictor for the development of subsequent anxiety. Previous studies have shown various situational and individual risk factors (e.g., unexpected deaths, kinship, social isolation) that contribute to an unfavorable bereavement outcome [63,64,65,66]. However, a significant amount of older adults seems to cope well with bereavement without experiencing an increase in psychological distress after the loss [67]. Similarly, Chou et al. [41] did not find the number of stressful life events, which included the death of a relative, to be a significant predictor for the incidence of anxiety disorders in older adults. The authors suggest that older adults may have acquired better skills to regulate their emotional states through life experience and are thus less influenced by such life events [41]. In sum, the results of this study indicate that bereavement per se does not predict subsequent anxiety in late life.

Finally, incidence of anxiety symptoms in late life is not predicted by basic sociodemographic factors (gender, age) or functional impairments. In line with our results, Samuelsson et al. [23] found no significant associations of sociodemographic and medical factors with anxiety in older adults. Previous studies with younger age groups mostly reported a lower risk of incident anxiety with increasing age [17,19,20] and male gender [19,49]. While incidence rates were higher in women compared to men in the present study, gender was not found to be a significant risk factor in multivariable analyses controlling for further variables. Gender differences can be explained through confounding with depression. Depression is commonly reported to be more frequent in women than in men [68,69]. Thus, when controlling for depression, gender may not be an additional predictor for incident anxiety symptoms. Furthermore, risk factors for the development of new anxiety episodes may differ over the lifespan. The influence of sociodemographic factors may decrease in later life as other risk factors, such as preexisting psychiatric disorders and cognitive functioning, become more important drivers of mental health problems in oldest-old individuals.

### 4.3. Strengths and Limitations

The strengths of the present study include the assessment of incident anxiety symptoms in a large cohort of oldest-old individuals. Oldest-old individuals are fairly under-represented in the field of mental health research. Previous studies have usually assessed incidence of anxiety in younger age groups or included only small subsamples of oldest-old individuals. The focus of the present study on individuals aged 81 years and older therefore sets this study aside and provides new information on mental health in this vulnerable and underrepresented subsample. Further, the present sample was recruited through GPs in six study centers across Germany, therefore increasing representativeness for community-dwelling adults in the oldest age groups. In consistency with the focus of the study on those age groups, symptoms of anxiety and depression were assessed using validated and age-appropriate instruments. The manifestation and reporting of anxiety symptoms may differ in old age specifically with regard to bodily sensations and physical health [25]. Physical symptoms are an important aspect of the assessment and diagnosis of anxiety. However, impairments in physical health are common in old age and may be downplayed or normalized rather than adequately recognized as part of anxiety by older adults and their physicians [25]. Thus, using a validated instrument, specifically developed to assess anxiety in old age is another strength of this study.

Limitations include the possibility of a selection bias due to predefined inclusion criteria and the exclusion of individuals with missing information on relevant outcome variables. Further, psychotropic drugs were not considered in the analyses and can therefore be regarded as a possible confounding effect. Regarding the primary outcome, anxiety was assessed at three single points in time. While this approach is commonly used in epidemiological studies, it may lack precision to truly reflect trajectories of anxiety, because episodes in between follow-up assessments may have been missed. Another limitation relates to the assessment of anxiety symptoms. Cases of anxiety symptoms were defined according to the GAI-SF and not according to a clinical diagnosis. However, as stated previously, the GAI-SF is an instrument specifically developed to assess anxiety in old age and has been shown to have good psychometric properties. Finally, individuals with dementia and individuals in nursing homes were not included in the present study. Thus, the presented incidence rates may be an underestimation of incident anxiety symptoms in the general population of that age group.

## 5. Conclusions

Knowledge about incidence of anxiety in latest life is scarce. Specifically large population-based cohort studies with oldest-old individuals are lacking in order to get a comprehensive understanding of risk factors of new anxiety episodes in this vulnerable age group. The present population-based study offers results on an understudied mental health topic in latest life. The development of new anxiety episodes is a frequent phenomenon in oldest-old individuals. Depression and subjective memory complaints are major risk factors of incident anxiety in that age group. The results of the present study indicate that anxiety functions as a response to the awareness of increasing memory difficulties independent of the presence of depression. However, the relationship of subjective memory complaints and anxiety in cognitively healthy oldest-old individuals is not well understood and needs further research. In comparison with existing literature, it appears that drivers of incident anxiety differ in latest life from younger age groups of older adults. Future studies should therefore address anxiety in old age stratified for different age bands. 

## Figures and Tables

**Table 1:** Sociodemographic Characteristics of the study sample at FU5.

	Study Sample (N = 702)
Age, years	
Mean, SD	86.4 (2.8)
Range	81–97
Gender, n (%)	
Male	236 (33.6)
Female	466 (66.4)
Education ^a^, n (%)	
High	108 (15.4)
Middle	213 (30.3)
Low	381 (54.3)
Marital status, n (%)	
Married	211 (30.1)
Widowed/divorced	445 (63.4)
Single	46 (6.5)
Living situation, n (%)	
Alone	371 (52.8)
Not alone	331 (47.2)
GAI-SF, mean (SD)	0.67 (0.77)
MMSE, mean (SD)	28.2 (1.4)
Depressive symptoms ^b^ n (%)	54 (7.7)
Recent loss experience, n (%)	202 (28.8)
Functional impairments, n (%)	
Mobility impairment	376 (53.6)
Vision impairment	159 (22.6)
Hearing impairment	337 (48.0)
Subjective memory complaints, n (%)	116 (16.5)

GAI-SF, short form of the Geriatric Anxiety Inventory; SD Standard deviation; MMSE, Mini-Mental-State Examination; ^a^ Classification according to the international new CASMIN educational classification; ^b^ Based on the Geriatric Depression Scale.

**Table 2:** Age- and gender-specific incidence of anxiety symptoms (GAI < 3 at FU5 and ≥ at FU6 and/or FU7).

	n	No. of New Cases	Sum of Risk Years	Incidence per 1000 Person-Years	(95% CI)
Total	702	77	1501	51.3	(41.2–64.1)
Men	236	19	509	37.3	(23.6–58.3)
Women	466	58	992	58.5	(43.2–72.4)
Age					
81 to 85	312	39	679	57.4	(41.8–78.6)
86 to 90	320	35	673	52.0	(35.7–72.8)
91 and over	70	3	149	20.1	(6.7–62.4)
Men					
81 to 85	127	16	273	58.6	(35.9–95.9)
86 to 90	89	3	194	15.5	(5.1–47.9)
91 and over	20	0	42	-	-
Women					
81 to 85	185	23	406	56.6	(37.7–85.2)
86 to 90	231	32	479	66.8	(47.2–94.6)
91 and over	50	3	107	28.0	(8.9–86.9)

GAI-SF, short form of the Geriatric Anxiety Inventory; No., Number; CI, confidence interval.

**Table 3:** Multivariable cox proportional hazards regression of time to incident anxiety for the overall sample and a subsample without depression at FU5.

	Overall Sample (N = 702)					Subsample without Depression at FU5 (N = 648)				
Variables at FU5	Incident Anxiety n/N	Multivariable HR (95% CI)	Wald F	SE	*p*-Value	Incident Anxiety n/N	Multivariable HR (95% CI)	Wald F	SE	*p*-Value
Age, every additional year	77/702	0.93 (0.85–1.01)	2.960	0.044	0.088	61/648	0.92 (0.84–1.00)	3.332	0.047	0.070
Gender										
Female	58/466	1				44/425	1			
Male	19/236	0.70 (0.42–1.19)	1.763	0.264	0.187	17/223	0.78 (0.44–1.39)	0.707	0.292	0.402
Depressive symptoms ^a^										
No	61/648	1				-	-	-	-	-
Yes	16/54	3.20 (1.46–7.01)	8.675	0.395	0.004	-	-	-	-	-
Recent loss experience										
No	54/500	1				42/463	1			
Yes	23/202	0.96 (0.58–1.62)	0.018	0.260	0.893	19/185	1.07 (0.61–1.88)	0.052	0.286	0.820
Subjective memory complaints (SMC)										
No	55/586	1				45/549	1			
Yes	22/116	2.03 (1.16–3.57)	6.202	0.285	0.014	16/99	1.92 (1.09–3.39)	5.170	0.287	0.025
Mobility impairment										
No	27/326	1				25/318	1			
Yes	50/376	1.42 (0.87–2.30)	2.045	0.245	0.155	36/330	1.43 (0.87–2.34)	2.049	0.249	0.155
Vision impairment										
No	55/543	1				42/505	1			
Yes	22/159	1.21 (0.72–2.05)	0.533	0.266	0.467	19/143	1.58 (0.93–2.69)	2.951	0.267	0.088
Hearing impairment										
No	45/365	1				33/331	1			
Yes	32/337	0.79 (0.47–1.34)	0.746	0.263	0.389	28/317	0.86 (0.48–1.54)	0.245	0.293	0.621
Depression*SMC	-	0.59 (0.18–1.94)	0.751	0.596	0.388	-	-	-	-	-

HR, Hazard ratio; 95% CI, 95% Confidence interval; SE, Standard error; FU, Follow-up; ^a^ Based on the Geriatric Depression Scale. The “*” is not an abbreviation here. It indicates an interaction term of Depression with SMC.

**Table 4:** Course of anxiety from follow-up wave 5 to 7.

FU5	FU6	FU7	N (%) ^a^	
OCT/2010-NOV/2012	JAN/2012- FEB/2014	JAN/2014- FEB/2015		
no anxiety	no anxiety	no anxiety	508 (72.4)	no anxiety
	no anxiety	anxiety	29 (4.1)	temporary anxiety
	anxiety	no anxiety	22 (3.1)	
	anxiety	anxiety	17 (2.4)	persistent anxiety

Based on the Geriatric Anxiety Inventory with a cut-off ≥ 3 for anxiety; FU5, FU6, FU7 = follow-up waves 5 to 7; ^a^ Missing data on either FU6 or FU7 for N = 126 (17.6%).

**Table 5:** Sample characteristics according to the course of anxiety from follow-up wave 5 to 7.

	No Anxiety (N = 508)	Temporary Anxiety (N = 51)	Persistent Anxiety (N = 17)
	Mean (SD)	Mean (SD)	Mean (SD)
Age (years)	86.45 (2.9)	85.8 (2.5)	86.0 (2.7)
	N (%)	N (%)	N (%)
Female sex	331 (65.2)	37 (72.5)	13 (76.5)
Education			
High	82 (16.1)	6 (11.8)	2 (11.8)
Middle	162 (31.9)	12 (23.5)	4 (23.5)
Low	264 (52.0)	33 (64.7)	11 (64.7)
Depressive symptoms	27 (5.3)	7 (13.7) *	5 (29.4) **
Subjective memory complaints	70 (13.8)	13 (25.5)	3 (17.6)
Recent loss experience	155 (30.5)	14 (27.5)	8 (47.1)

Based on the Geriatric Anxiety Inventory with a cut-off ≥ 3 for anxiety; no anxiety, GAI < 3 at follow-up waves 5 to 7; temporary anxiety, GAI ≥ 3 at either follow-up wave 6 or 7; persistent anxiety, GAI ≥ 3 at follow-up waves 6 and 7; * significant differences between ‘temporary anxiety’ and ‘no anxiety’, chi2 (1, 559) = 5.739, *p* = 0.027; ** significant differences between ‘persistent anxiety’ and ‘no anxiety’, chi2 (1, 525) = 16.688, *p* = 0.002.

## Data Availability

The data that support the findings of this study are available on request from the corresponding author. The data are not publicly available due to privacy or ethical restrictions.

## Supplementary Materials

The following are available online at https://www.mdpi.com/article/10.3390/ijerph182312786/s1, Table S1: Sample characteristics of excluded and included participants at FU5.
