# Supplementary material for: Incidence of Anxiety in Latest Life and Risk Factors. Results of the AgeCoDe/AgeQualiDe Study
Source: Int J Environ Res Public Health. 2021 Dec 3;18(23):12786. doi: 10.3390/ijerph182312786 (PMC8657712; doi:10.3390/ijerph182312786)
Supplement: Supplementary file 1 [file ijerph-18-12786-s001.zip › ijerph-1478939-supplementary.pdf]

SUPPLEMENTARY MATERIAL

Table S1. Sample characteristics of excluded and included participants at FU5.

	excluded individuals (N=640)	included individuals (N=702)	p-value*
	Mean (SD)	Mean (SD)	
Age (years)	87.2 (3.47)	86.4 (2.82)	<.001
	n (%)	n (%)	
Gender, female	453 (70.8)	466 (66.4)	.047
Education			
High	59 (9.2)	108 (15.4)	<.001
Middle	188 (29.4)	213 (30.3)	
Low	393 (61.4)	381 (54.3)	
Marital status[a]			
Married	169 (26.5)	211 (30.1)	.332
Widowed/divorced	421 (66.0)	445 (63.4)	
Single	48 (7.5)	46 (6.6)	

SD Standard deviation; FU5 follow-up wave 5; *based on Chi2-test or Mann-Whitney-U-Test for independent samples as appropriate, [a]Missing data for excluded individuals: N=2.
